# This Number of the Journal

**Published:** 1916-04

**Authors:** 


### This Number of the Journal.

The April number of the TEXAS MEDICAL JOURNAL offers a demonstration of what women are doing in the practice of medicine in this country and especially in Texas. Where women have gone into medicine with serious purpose they have had remarkable success, almost without exception. The papers that are presented in this issue, written by medical women, show that the women are just as progressive and alert in the profession as the men. But for the captions or signatures to the articles no one would ever know that they were not written by men. Why should that question ever be raised, though? Women are proving every day that they are prepared to successfully cope with the men in most of the professions and vocations in life. The strongest and ablest men are conceding this. They are no longer considered as rivals in the professions, but as associates and co-laborers. While this issue of the JOURNAL is almost altogether the work of women practitioners, it is not intended to interest women merely, but will be found as instructive and helpful to the medical men as any number that has ever been issued. Women have been contributors frequently before, and this issue merely assembles a number of their contributions in order to call attention with some force to the fact that women not only are practicing, with much success, but that they know how to present their studies and conclusions in readable form.

---

Dr. and Mrs. Collard of Mumford left recently for an extended trip to New Orleans, where the doctor will attend lectures.

